# COVID-19 Governance in the Event Sector: A German Case Study

**DOI:** 10.1007/s41125-022-00088-6

**Published:** 2022-12-20

**Authors:** Malte Schönefeld, Patricia M. Schütte, Yannic Schulte, Frank Fiedrich

**Affiliations:** Lehrstuhl für Bevölkerungsschutz, Katastrophenhilfe und Objektsicherheit (Chair for Public Safety and Emergency Management), University of Wuppertal, Wuppertal, Germany

**Keywords:** Qualitative research, Event safety, Governance, COVID-19, Neo-institutionalism

## Abstract

The article reflects on the stop-and-go procedures of re-opening the event sector under pandemic circumstances in a case study for difficult political and administrative governance, confusing regulations and systemic irritation. The focus lies on the addressees of restricting regulations, i.e. event industry and in particular event organizers who have to deal with requirements from different event stakeholders. It is our aim to trace their strategies and identified margins of manoeuvre in order to re-enable events under inconvenient surrounding conditions. In times of COVID-19, major events are under general suspicion as enablers for “super spreading” or “mass contagion”. One of the major business sectors in Germany—the event sector—was among the very first that was forced to shut down and among the very last, that could re-open again. This has not only economic but also social impacts: events as social settings and contexts fulfil important societal functions. They enable social exchange, cultural innovation, and political participation and provide socio-psychological relief. The contribution of events to these elementary societal functions was strongly limited in the pandemic. Although event approving authorities and event organizers in collaboration with their service providers work intensely to re-open events under hygienically safe conditions, lastingly convincing re-opening concepts have not yet been identified. The federal system in Germany, the diversity of applicable regulations, expected measures and outcomes, the dynamics of the situation, and resulting short-term changes in legal conditions lead to a variety of concepts and measures, which differ depending on location, event, persons involved, etc.

## Introduction

The COVID-19 pandemic has hit the event industry hard. It has been more or less at a standstill since 2020. One of the top 10 economic branches with a revenue of 129 billion Euro and around 1.5 million people at work in 2019 (R.I.F.E.L. 2020) in Germany—the event sector—was among the very first that was forced to shut down and among the very last that could re-open again. Since then, rules and regulations for events were oscillating in high frequency between these two poles, which negatively affects planning reliability for event organizers. Although event approving authorities and event organizers in collaboration with their service providers worked intensely to re-open events under hygienically safe conditions, lastingly convincing re-opening concepts have not yet been identified. The federal system in Germany, the diversity of applicable regulations, expected measures and outcomes, the dynamics of the situation, and resulting short-term changes in legal conditions led to a variety of concepts and measures, which differed depending on location, event, persons involved, etc. Going into the beginning of 2022, the industry was characterized by stop-and-go procedures of re-opening the event sector under pandemic circumstances. The situation still seems difficult and unpredictable. Nevertheless, the many attempts by event organizers to launch concepts for safely implementing events again speak for a relentless quest to revive the event industry. This raises the question of the basis on which the approaches, re-opening concepts and measures were and are actually taken. What requirements from different stakeholders did the event organizers face? How did they deal with the multiple demands and expectations? What kind of logic did they follow in their decisions (efficacy, efficiency, economical aspects, acceptance, rationality and/or legitimacy)? The focus here lies on the addresses of restricting regulations, i.e. event industry, in particular event organizers. It is our aim to trace their strategies and identified margins of manoeuvre in order to re-enable events under inconvenient surrounding conditions.

In an attempt to answer these questions, we aim to trace the relevant requirements that have had an impact on the event organizers’ decisions regarding the planning of events as well as their logics behind, based on empirical data obtained in first-hand examinations in the NORMALISE research project (see Sect. 4). From a theoretical point of view, we refer to neo-institutionalism and assume that organizers do not orient themselves solely to economic, acceptable and feasible solutions, but to what seems rational and is expected, if not demanded, by the environment. Re-opening at any price and writing everything necessary into the concepts seems to be the main claim of the organizers. In other words, it is all about legitimacy in order to realize their own events and possibly secure their own survival as an organization.

## Background: Event Approvals and COVID-19

Whether a major event like a music festival, a carnival or a (mega) sports event can take place or not depends largely on the assessment on the safety of the event. Event safety in Germany is part of regulations at the federal state level, at which interestingly, in most federal state law, legal definitions of major events are missing. Unfortunately, progress in event safety is highly driven by critical incidents and failures (Challenger and Clegg 2011; Mair and Weber 2019; Vendelø 2019). Especially since the Love Parade incident 2010 (Helbing and Mukerji 2012), flaws and shortcomings in German event safety became highly visible. The matter was taken up at the national level: Event safety research projects were initiated, and a working group for “Aspects of major events relevant to civil protection” within the portfolio of the Federal Ministry of the Interior began to address the establishment of national standards related to event safety (Coellen and Franke 2014). One example for these efforts is a common definition for the object at risk, “major event”, illustrating the basal level at which the discussion was conducted in Germany a decade ago. A research project under participation of the German Federal Office for Civil Protection and Disaster Relief aimed to fill that definitional gap only recently and established a common understanding among most stakeholders that we adopt in this article:

“Events with an increased risk potential due to the nature of the event or at which the number of visitors is higher than one third of the population [of the host city] or higher than 5000. They require official approval as well as qualified cooperation between the authorities and organizations with safety and security tasks (AOS), the event organizers and other parties involved.” (BaSiGo 2015: glossary entry “Großveranstaltung”, translated by authors)

Further developments and the necessary attention to the area of event safety have followed in the aftermaths of several incidents and near-misses, becoming manifest in technical, organizational, and personal measures against dangers like crowd dynamics/stampedes, terrorism, amok, weather hazards, and other threats. Event safety concepts became more comprehensive and professional, largely by the expectations of the regulatory agencies, which are responsible for event permissions at the local level.

However, questions of public health in the event context did not raise comparable attention. Although infection risks are inherent to the characteristic of events as gatherings of many people, these risks were not given particular regard. As our interviews show (see Sects. 3–5), the event sector was catched flat-footed when the pandemic threatened their business continuity, with little knowledge and high insecurity about how to conduct safe events under pandemic circumstances. Consequently, events were prohibited until further notice. This puts organizers under pressure. They were and potentially will again be confronted with a wide variety of requirements that they had and will have to meet in order to re-open their events and generate turnover again.

Over the course of the pandemic, the event sector was experiencing a rollercoaster ride in terms of what is allowed and what is not. Between periods of total shutdown of event operations and phases of a delusive back-to-normal feeling, there were transition phases characterized by several limitations, e.g. in terms of rules of conduct (“No singing!”, “Keep distance!”) or in terms of maximum permitted audience (limitation of capacity).

**Fig. 1:**
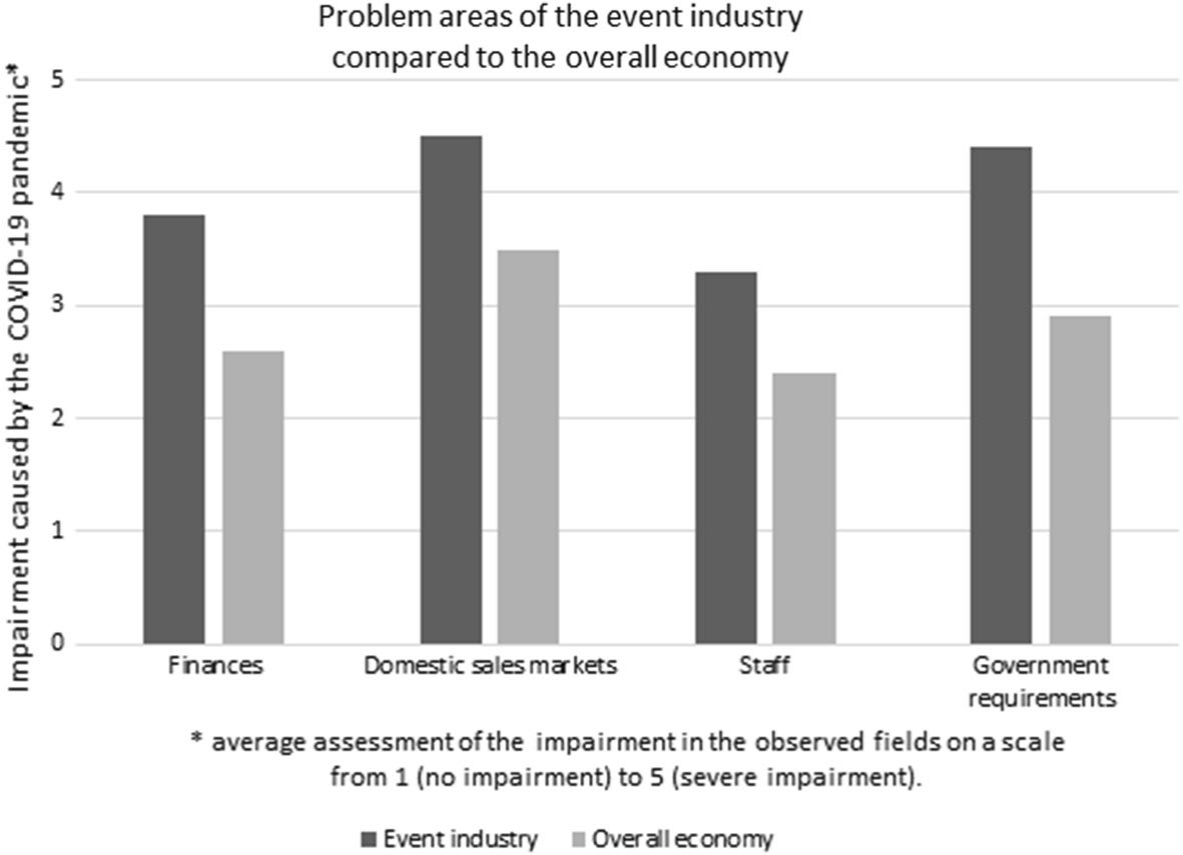
Problem areas of the event industry compared to the overall economy (Sauer and Wohlrabe 2021) (translated by the authors)

The situation left its marks in the event industry. The sector feels more impaired by the COVID-19 pandemic than the overall economy. As Fig. 1 shows, the ordinances, regulations and requirements imposed by “the government” and subsequent official actors are an area of very high concern for the event industry.

Events fulfil important societal, economic, and cultural functions. The fragile situation impaired this huge potential of events. In addition to the social significance, the re-opening of events was also important for the industry itself. This economically hard-hit sector struggled to keep itself and its workforce alive. Although two years did pass since beginning of the COVID-19 pandemic, a convincing re-opening concept is still to be found. In the following, we look at the ordinary event approval procedure before we introduce our theoretical viewpoint.

### The Event Approval Process

**Fig. 2:**
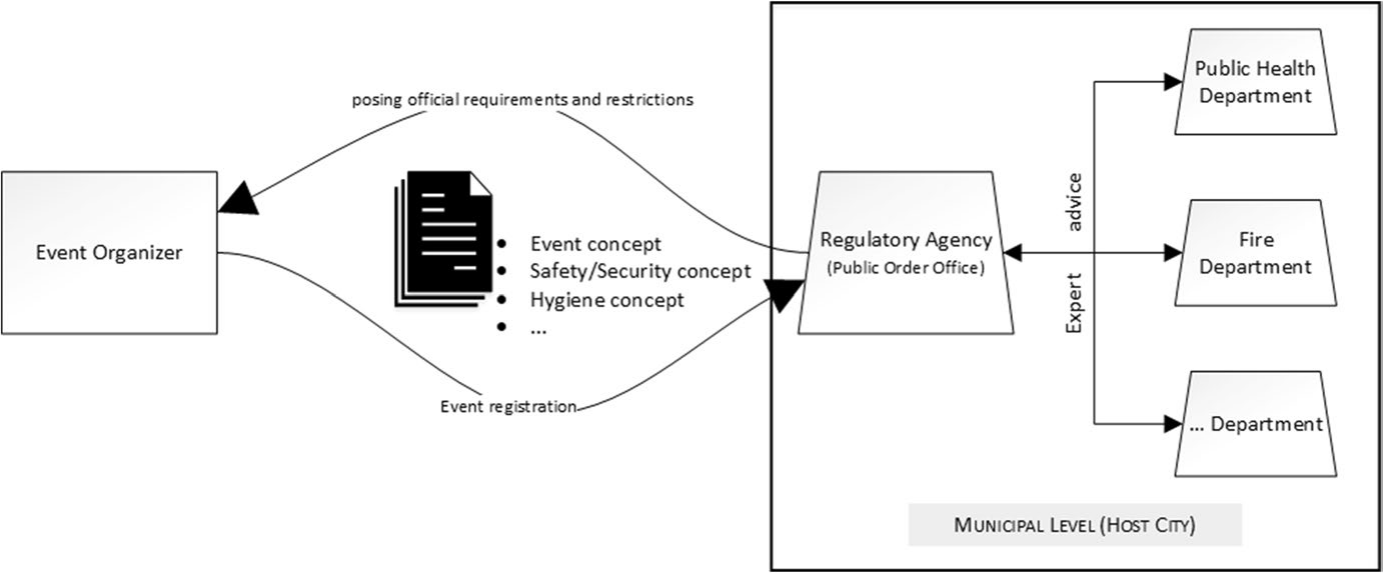
Event approval process in Germany (own illustration)

In Germany, an event organizer with the intention of holding a certain event is seeking approval for its conduction. For this approval, a registration including an overall event concept as well as a concept for safety and security is handed in at the approval or regulatory agency that is part of the local layer of governance (Fig. 2). Only during COVID-19, an additional hygiene concept often became a regular supplementary document in which the compliance to existing rules for infection prevention was laid out.

In most cases, the regulatory agency is identical with the public order office (*Ordnungsamt*) which may consult other specialist public authorities like fire or public health services (*Feuerwehr*, *Gesundheitsamt*) for expert advice in the approval procedure. The regulatory agency collects and sums up all inputs from the departments consulted and may subsequently request modifications to specific details of the concepts.

As Fig. 2 indicates, the event approval process involves several other important actors, which will be presented subsequently in order to explain the various organizational logics (Greenwood et al. 2009; Thornton 2004), e.g. intentions, values, rules, etc., that need to be addressed by event organizers in the approval process.

### The Event Approval Stakeholders

*The* *event* *organizer* The organizer is responsible for the safety of the event. An organizer is a natural or legal person who carries out an event on his own responsibility. Organizers can pursue both commercial and non-commercial interests; they can work professionally and full-time or part-time on a “hobby basis”. The size of an event is not an indication of the professionalism of the “organizer”. In Germany, there are no requirements for “the organizer” in terms of qualification and training. There is no standardized concept for “the organizer” with clearly defined roles, training profiles, operational requirements, etc.—just as “the organizer” is not the only role to be filled in this function. The organizer is also an entrepreneur and/or employer in the sense of the occupational health and safety laws and the accident prevention regulations. The event organizer presents its intention to organize an event in a given place to the responsible regulatory agency. The complexity of the registration process may vary.

*The* *regulatory* *agency* The regulatory agency is always bound to existing law. As a public body, any intervention must happen on a legal basis. Legal sources (e.g. directives, norms) with regard to event safety are regularly located on the federal state level (Löhr 2018). Their scope ranges from technical regulations to organizational processes like necessary qualifications of safety personnel, etc.

Depending on the outline data, the regulatory agency may bind its approval to conditions, such as putting concepts and measures for safety and security in place, and nowadays, as a new requirement, also for hygiene. This process may be iterated and also include discussions, site inspections, etc. The diversity of applicable regulations, expected measures and outcomes, the dynamics of the situation and resulting short-term changes in legal conditions lead to a variety of concepts and measures, which differ depending on location, event, persons involved, etc.

*The* *public* *health* *office* Public health offices are (with the exception of few federal states) part of the municipal level. Their main task is the implementation of the Infection Prevention Act (*Infektionsschutzgesetz*), which regulates the cooperation and collaboration of federal, state and local authorities. It defines reporting obligations and individual measures for the prevention of transmissible diseases, e.g. the obligation to provide proof of vaccination. For this reason, it was essential for event organizers to draw up a hygiene concept (in addition to the usual safety concept). The measures taken (e.g. air filters, capacity restrictions, mandatory testing, contact tracing and vaccination verification) are specified here, in compliance with the current regulations of the respective federal state in which the event is to take place. To date, there are hardly any standards in this area, which is why hygiene concepts vary widely. But since the pandemic, public health offices therefore find themselves under the spotlight of public attention by shifting from a “shadowy existence” as public health supervisor to a key crisis management authority (Kuhlmann and Franzke 2021). They have taken a major role in the event approval process as the specialized infection prevention agency in the municipal framework.

*Public* *authorities* *and* *organizations* *with* *security* *tasks* Within their realm of expertise, police and fire departments are often included into the event approval process, e.g. for reasons of public safety, fire protection, traffic control, etc.

Not part of the official approval process, but clearly indicated in our interview findings is the role of local politics. As shown later, local politics tend to favour re-opening over continued restriction and thereby influencing the administrative branch in the local governance framework. The final approval, however, is based on a discretionary decision of the regulatory/approval agency in compliance with federal state law.

Event-goers as the addressees of most of the safety, security and hygiene measures also constitute an important influential factor. Many measures require active cooperation and interaction, which is why compliant behaviour of event-goers is necessary for safety and security. We therefore assume that organizations are guided by their perceptions of the acceptance of measures by their visitors/customers in order to maximize their impact.

## Theoretical Perspective on Event-Related COVID-19 Governance in the Event Industry with a Focus on Event Organizers

As already briefly mentioned in the previous section, several triggers have a potential impact on developments in the event industry and particularly on decisions of event organizers as a central actor group in this field. They have a special role because they have to meet many requirements if they want to obtain legitimacy, i.e. a permission to implement an event. Their decisions regarding events are strongly influenced by different environmental factors. The following examples serve to illustrate which influences and requirements affect organizers.

Since 2010, it was, e.g. failures and critical incidents, but particularly terrorist threats that shaped considerations and actions for major events. In this context, practitioners reported that terrorism at major events tended to shift other critical scenarios such as extreme weather events into the background of security discourses and concepts. This is evident at various so-called *field-configuring* *events* (Lampel and Meyer 2008; Wooten and Hoffman 2016) such as conferences and workshops, where field representatives regularly meet and exchange ideas. In Germany, too, such *trigger* *topics* seem to have a lasting effect and led to legal and normative evolutions that forced event organizers to develop comprehensive safety concepts. They became legally anchored necessities for approvals, which have to be agreed upon by all relevant event stakeholders. This establishment of agreement involves various authorities and private security providers, which are all part of the field of event safety. As organizational behaviour can be seen as embedded in social and institutional contexts, it is influenced by different institutional logics that influence the organizations of such a field. These are logics of action through which institutions—family, market, the bureaucratic state, democracy, religion, professions and society—shape, change, stabilize, etc., individual and organizational action, as Friedland and Alford (1991), later also Thornton (2002, 2004) and Thornton and Occasio (2008) describe it. Institutional logics offer some kind of orientation for organizations in terms of “assumptions and values, usually implicit, about how to interpret organizational reality, what constitutes appropriate behavior, and how to succeed” (Thornton 2004, 70). They define the possibilities for action of an organization. The organization, in turn, is influenced by different logics, which can sometimes conflict. Therefore, it combines and deals with them in its specific organizational logic, which means, “a composite expression of a range of institutional logics localized in time and space” (Spicer and Sewell 2010, 7; Friedland and Alford 1991; Thornton and Ocasio 1999). In the context of the event industry and in particular event safety as an essential part of it, this means security and safety aspects, rules and practices, for example (Greenwood et al. 2009; Thornton 2004), which are mutually agreed on in a safety concept. Organizers must orchestrate this process well so that the authorities involved support the safety concept (see below). This is a prerequisite for the final legitimization of the event implementation by the approval authority (see Sect. 2). However, the organizers also pursue market-based premises of profit making, which, for example, keep the budget for safety within limits.

In addition, non-binding regulations that had been in place for years like the ‘Model Ordinance on the Construction and Operation of Places of Assembly’ (German regulation: *Musterverordnung* *über* *den* *Bau* *und* *Betrieb* *von* *Versammlungsstätten*, *MVStättVO*) were translated into federal state law equivalents and implemented (Löhr 2018). To fill the required concepts with life, i.e. concrete content, guidelines and orientation frameworks were developed such as the security guidelines for major outdoor events issued by the Ministry of the Interior and Local Government of the State of North Rhine-Westphalia (MIK NRW 2012) or the *BaSiGo* *Guide* (BaSiGo 2015) which developed from a publicly funded research project. Concepts implemented according to such orientation frameworks and guidelines, established themselves as benchmarks of professionalism for event organization. Even though these may not be binding regulations, their consideration by event organizers is seen and ultimately expected by many authorities as a characteristic of a professional event. Additionally, there are also mimetic effects, for example, between various event organizers, which result from regular meetings for the exchange of information on event management and event safety and security, as well as from mutual observation. An important pioneer in the area of professionalization is the German Football Association (*Deutscher* *Fussball-Bund*), which has found some imitators, e.g. in the training courses developed for private security personnel and event stewards. Changed attitudes towards security and safety and an increased sensitivity towards subjective (in)security feelings and expectations of visitors—as examples of environmental factors—seem to have gained increasing influence as an important variable in decisions about measures on the part of organizers and security actors at major events (keyword: “security theatre”) (Felten 2004; Schütte et al. 2021). To put it concisely, (critical) incidents, resulting regulations, changing expectations of diverse stakeholders, different logics, actions of and interactions with other stakeholders influence the event organizers’ decisions (DiMaggio and Powell 1983, 1991; Greenwood et al. 2009; Scott 1991; Wooten and Hoffman 2016).

**Fig. 3:**
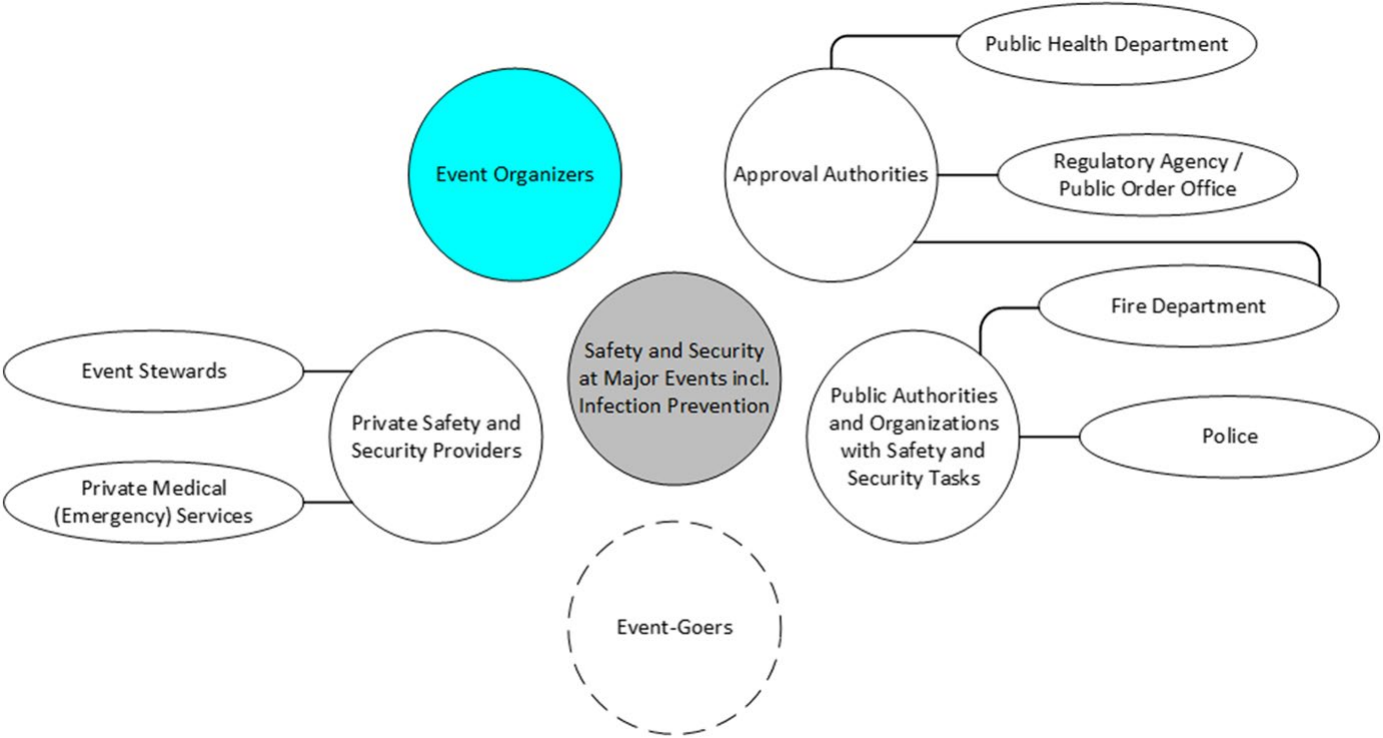
Key stakeholders for safety and security at major events (own illustration)

The COVID-19 pandemic represents a *caesura* in this context, as it sets a completely different issue alongside classic event security and safety. The classic safety and security requirements from respective stakeholders for event organizers were supplemented by additional quite different requirements from additional stakeholders (i.e. health departments). As a result, event organizers were under even more pressure than before to decide which requirements they must, can and want to fulfil. In order to decide sensibly they had to weigh economic, security-, safety- and hygiene-related aspects as well as acceptance- and legitimacy-promoting arguments, etc., and had to prioritize the demands of other stakeholders (health department, public order department, police, fire brigades, event-goers, etc.; see Fig. 3).

We assume that for event organizers, the legitimacy of their events was at the forefront of their decisions. By granting permission to hold events, they were able to (re)open up economic profit opportunities, avert further drastic losses and secure their survival. This understanding of legitimacy corresponds to classical definitions as used in neo-institutionalism, which means, it “is a generalized perception or assumption that actions of an entity are desirable, proper, or appropriate within some socially constructed system of norms, values, beliefs, and definitions”. (Suchman 1995:574). One way of dealing with many different, sometimes conflicting requirements is *decoupling* what actually happens in an organization from formal structures (Meyer and Rowan 1977). The structures thus take up expectations of the environment without actually translating them into actions. They primarily fulfil a symbolic function. This refers to so-called legitimacy facades (e.g. Meyer and Rowan 1977; Suchman 1995). This is closely related to new challenges for organizers that arose from the pandemic situation:

- The public health departments as new actors in the event field, knew little about it, and were trying to protect themselves legally. This often created a multitude of requirements that could hardly be reviewed by an agency staff tied up in the pandemic.
- Since COVID-19 was all-determining situation, health departments became central actors and their professional logics dominated over other actors less central in the situation. Hygiene requirements were put before safety and security requirements. As a result, event organizers implemented what was assumed rational by stakeholders in times of the COVID-19 pandemic, such as comprehensive concepts and measures for infection control and hygiene in order to obtain permission for the re-opening of their event.
- Nevertheless, at the same time, the requirements and professional logics of *classic* authorities remained relevant for the legitimization of organizers and the approval of events. They, too, strove to secure themselves legally and to demand the appropriate safety measures and corresponding resources to do so in terms of fire protection, emergency rescue, paramedics, etc.). However, those actors were not responsible for hygiene requirements and were therefore not very interested in them. There was no coordination whatsoever between hygiene concepts on the one hand and safety and security concepts on the other hand, which means that organizers have to use resources twice.

To examine this more closely, we turn to the question of how event organizers deal with the different requirements, some of which contradict each other and some of which interact with each other.

Based on the considerations outlined and on interview statements of key stakeholders (see Fig. 3), we are investigating the logics of event organizers and the multiple requirements they are dealing with as bases for decisions regarding hygiene concepts and infection control measures for a supposedly safe re-opening of major events. In the following, we will go into more detail on the objectives, the methodological approach and the empirical data repository of the aforementioned project.

## Methodology

In order to investigate how event organizers try to reach conformity with the requirements and how they use the regulatory margins of the COVID-19-related hygiene regulations, empirical data from our publicly funded German research project NORMALISE are used here. In the following, we will focus on data obtained in expert interviews with representatives from key stakeholder groups, and in field observations at events.

*Expert* *interviews* From July to September 2021, eleven semi-structured expert interviews were conducted (see Table 1). The experts were recruited from the above-mentioned stakeholder groups (see Fig. 3). The choice of interviewees resulted from an investigation about actors who were actively involved in event approval processes under COVID-19 conditions as well as the event organizers themselves. These contacts were also used for field access interviews. Here, the stakeholders were able to additionally name whom they considered relevant for the approval process for events.

**Table 1:** List of interviewees

Type	Interviewee	Date
Private entity	Organizer 1 (sports)	29 July 2021
Private entity	Organizer 2 (music festivals)	02 August 2021
Private entity	Organizer 3 (various)	19 August 2021
Private entity	Private event safety 1	13 July 2021
Public authority	Approval authority 1	16 August 2021
Public authority	AOS 1 (Police)	05 August 2021
Public authority	AOS 2 and 3 (Fire Department, joint interview)	09 August 2021
Public authority	Health authority 1	01 September 2021
Other	Independent expert 1 (for hygiene)	20 July 2021
Other	Independent expert 2 (for event safety)	29 July 2021
Other	Independent expert 3 (for event safety)	02 August 2021

The interviews took place at a time when major events in Germany were temporarily realizable under COVID-19-related restrictions. Thus, on the one hand, it was possible to look retrospectively at events that had already been planned and carried out. On the other hand, it was possible to look prospectively at (coordination) processes of events that were yet to take place. Thematically, the experts were asked about the pandemic-related alterations of the event approval process, about the development of concepts for hygiene/infection prevention, and about the implementation of hygiene measures, as well as the need for guidelines and an orientation framework for the planning and execution of events under COVID-19 conditions. In this context, strategies and coordination processes of stakeholders and margins for manoeuvre were also discussed. The interviews were subsequently transcribed and anonymized. For the evaluation, the thematic qualitative text analysis (Kuckartz 2014, pp. 69ff) was chosen which is a deductive–inductive approach. In a first step, we coded the expert interviews using deductively developed categories based on the structured interview guideline and theoretical constructs from past research projects (e.g. mutual perception of the actors). The deductive categories used are as follows:

- Framework/environment (applicable COVID-19-specific event sets of rules) that have an impact on the actors.
- Which regulatory margins remain, are used or are desired.
- The mutual perception of the actors.
- As well as their own perception and understanding of their roles.

As a second step, the iterative inductive coding process on the material then allowed the changes through COVID-19-specific aspects in the approval process to be explored in detail. Here, the process-oriented approach (Zilber and Meyer 2022) is used to consider the temporal dynamics (categories: framework/environment and which regulatory margins remain; see Sect. 5.1) in regulations for events and the impact on event management from the organizer’s perspective. In a final step, the main statements were then extracted, compared and analysed with a focus on event organizers and their dealing with various environmental requirements.

*Field* *observations* As part of the NORMALISE project, semi-structured field observations were made and documented at five different events from September to December 2021 (see Table 2). These included two outdoor sport events, two indoor music events, and an outdoor Christmas market. The observations were focused on the interactions of visitors and event staff with infection control measures. Furthermore, it was possible to participate in a coordination meeting between authorities and the organizer related to one of the outdoor sport events. In the course of the observations, side talks with event organizers about strategy in terms of approaching challenges in planning and implementation took place. Observation logs were used as a supplementary source of insight into how interorganizational negotiations took place and how the hygiene concepts were put into practice.

**Table 2:** Description of conducted field observations

Event type	Venue type	Date	No. of visitors (approx.)
Sports event 1	Outdoor	Aug. 2021 (1 day)	5.000
Sports event 2	Outdoor	Nov. 2021 (1 day)	26.000
Music festival 1	Indoor	Oct. 2021 (2 days)	4.000
Music festival 2	Indoor	Nov. 2021 (2 days)	4.000
Christmas market	Outdoor	Dec. 2021 (3 weeks)	n/a (no count)

The findings from the empirical data collection as outlined above are presented in the following section.

## Findings

To contextualize the following findings, it helps to briefly recall the organizational logics of the actors and groups of actors in the approval process (see Fig. 2). Organizers are interested in the success of an approved event. They value (economic) success, stable framework conditions in planning and execution, and customer satisfaction. The licensing authority and its related public partner organizations, such as the health department, pursue health and safety enforcement based on applicable law and the framework of the event being licensed.

The explorative interviews revealed interesting phenomena with regard to adapting to the new situation in the context of the re-opening of major events. As already described above, we consider statements from various key stakeholders in the event approval process in order to explain the challenges and constraints which event organizers face from different perspectives.

- Event organizers suffer from planning uncertainty due to rapidly changing regulations and difficult medium-term foresight that also lets event organizers in partnership with their safety and security contractors face difficulties when communicating compliance of event-goers to non-pharmaceutical interventions (NPI) due to heterogeneity of NPI between events and between event venues (5.1).
- The relationship between event organizers and the new player “public health department” has not yet properly settled due to its struggle for accepting its expertise and power. Given this, plus the lack of resources of public health departments to consistently and credibly translate their regulatory power into action, margins of manoeuvre (resp. grey areas) open up for event organizers (5.2).
- Delegation of pressure: Local politics favour clean re-opening and delegates responsibility to its administration which in turn translates this pressure into harsh requirements towards event organizers (5.3).

In the following, these findings will be presented in detail:

### Ever Changing Circumstances, Diversity of NPI Regulations, Lead-Time and Planning Reliability

Regulations for the conduction of events under pandemic circumstances changed over and over since the total closure of events in early 2020. The concerns of event organizers are not so much the constraints themselves that come with pandemic-related ordinances but their repeated altering and the short periods of ordinance validity. This includes the overall uncertainty whether major events will be allowed at all and, if yes, which circumstances need to be considered in the respective “hygiene concept”. One event organizer describes his situation at the end of *July* *2021*, when s/he was currently planning an event for *fall* *2021*, as a leap into the dark:

“In [a particular German federal state] there is simply no corona control ordinance that already includes October or November, so that the eventual approval authority could say, ‘At this point in time, what you’re proposing to me here makes total sense’. Because I do not doubt at all that they understand that—they don’t lack the expertise to understand that. I do not think they do. They match that with the appropriate legal situation that exists and say at this point in time, ‘Great’. However, the next corona protection ordinance […] will come at the end of September. What will be in it? The responsible approval authority does not even know now—two months before. (…) If I put myself in the position of this person, I can understand that he says: ‘Well, I don’t know what the entire requirement is which is given to me by my higher authority, namely the responsible federal state authority. That’s why I can’t tell you now whether the concept will then, if the event is to take place, still be approvable.” (Organizer 2, item 30).^1^

Both event organizers and local authorities are left in darkness, and it is rather the federal state level at which the criticism is directed. Although this level has to make decisions under uncertainty due to the dynamic situation, it is ultimately the one where the altering ordinances and regulations originate. Another event organizer joins the empathic attitude towards local public servants:

“At the end of the day, the lady from the approval authority also said to me this morning, ‘I didn’t get the ordinance any earlier than you did. You got it Tuesday afternoon, and I got it Tuesday afternoon. And at that moment the storm of questions already broke over me.’ She is not to be envied.” (Organizer 3, item 18)

According to interviewees, this created an atmosphere of uncertainty, since major events often require some weeks or months of lead-time in order to plan properly. It is difficult to manage otherwise. Even if the overall regulations may eventually allow the conduction of an event, the organizer regularly needs to build up contractual relationships with service providers which he/she may have a hard time to arrange if there is a serious economic risk for the organizer itself or its contractors:

“If the general condition is, ‘work first, but we don’t know exactly whether it will take place, because we don’t know whether we will get an approval in the current pandemic situation. And if that’s not the case, then it’s also difficult for us to pay you, because we don’t have any income either’, then, of course, no one will start working. On top of that, these companies, like the organizers, are also working short time.” (Organizer 2, item 19)

Approval authorities, also affected by the altering regulations and short validity periods, are well aware of the organizers’ dilemma:

“That’s why I think they’re playing it safe at the moment and prefer to postpone the whole thing until next year; otherwise they might just waste money and time for organizing [the event].” (Approval authority 1, item 5)

Those who “play it safe”, in the words of this approval authority staff member, are not the big commercial players but the small and local non- or low-profits:

“For all these semi-professional organizers of street festivals and alike, as we have here in [CITY], I think they just don’t do that. That is because it is probably just a bit too big for them. Creating hygiene concepts, going into planning without ultimately having a guarantee that it can take place in the end and so on.” (AOS 2 & 3, item 19)

The professional organizers of comparably large events in sports (here: professional soccer) face very different circumstances in this regard as they acknowledge due to more resources and expertise:

“I think we [...] have the advantage that soccer is, on the one hand, highly organized, also well-staffed. That’s certainly a big advantage, and I’d say that the financial possibilities and the personnel expertise have of course also enabled them to develop a good precursor model, so to speak, and to approach the matter with a great deal of precision, I’d say, and perfectionism. So many people put a lot of thought into how they would then implement it operationally on the ground. Of course, this is much more difficult for other sports, which are also based on volunteers.” (Organizer 1, item 33)

However, there is not only limited planning reliability on the time scale, there is also a spatial dimension: As of March 2022, it is up to the individual local approval authority to decide whether proposed measures, written down in a “hygiene concept”, are sufficient or not. There is no common standard for the use of non-pharmaceutical interventions at major events that is in place all over Germany, of course due to its federal structure. However, it is not uncommon for events to “tour” through different cities, and federal states. The more individual rules they encounter, the more onerous it becomes for them:

“In wishful thinking […] an organizer also knows that if I now plan a nationwide tour, then I will find similar or at least similar conditions in Bavaria as in North Rhine-Westphalia and as in Berlin. However, that is not the case at all.” (Organizer 3, item 47)

Moreover, even in the same city, in the case of similar sports events of the same organizer in the same indoor venue, one and the same licensing authority may come to different conclusions, from which the assigned steward service could not conclude what the differences in regulation were based on if not interpretation:

“At the first event, it was immensely important that the temperature was taken at everyone’s entrance at the arena. At the second event, it did not matter at all. So there is no uniformity in the approval authorities either, the same approval authority by the way. [...] You can see that this uniformity of measures does not exist. [...] There are guidelines, but they are interpreted or demanded differently within the same authority. That is my experience.” [Private event safety 1, item 22]

The assessed situation is far from standardization. This heterogeneity of requirements leads to a heterogeneity of measures, which has a certain implication for the compliance of visitors as well. In addition to increased effort for the organizer, changing rules and obligations can also lead to confusion or lack of understanding on the part of event guests, or—as one public safety officer puts it—heterogeneity impairs compliance:

“Nothing is more unfortunate than when I attend two events and one is like this and the other is completely different. That always leads to a dwindling acceptance” (AOS 1, item 36)

Acceptance and compliance of event visitors are important when reflecting the positions, debates, and interests of approval authorities as well as of event organizers. They are the addressees of most of the measures involved. It is them who shall be protected from infections. However, similar to most technical, organizational, or personal measures of “classic” event safety, the event visitors have to play along for the measure to have an effect. Furthermore, comparable to classic event safety measures, measures must be considered as sense-making to the visitor in order to be accepted and to be complied to. Event organizers know this mechanism well as it stands in contrast to the facades they set up in extensive hygiene conceptualization:

“The purpose should be to take measures where one is sure that they will have a certain acceptance. (...) It makes no sense that it only looks nice on paper if you know (...) that it will not work in practice” (Organizer 2, item 37).

In sum, on the part of the approval authorities, decisions are probably lagged because the legal and political conditions require it (coercive logics; see above). On the part of the organizers, this possibly leads to an—at least superficial—*overachievement* of the requirements in order to signal conformity with the expectations and specifications:

“So first of all, that was not requested, that was proposed by the [event organizer] on its own, the 3G regulation. It was not the health department. It goes without saying that the health department does not say at this point, ‘You don’t need all this, you can do without it’. It just gives everyone a better feeling somewhere when overachievement is achieved.” (Approval authority 1, item 41).

However, there are also organizers who take the situation as an opportunity and who already think ahead to professionalize their concept design with regard to the integration of safety and hygiene and to position themselves more flexibly. However, in this context, it becomes clear how different the logics of the various stakeholders within the field are and how little each of them incorporates the perspectives of the other.

### Relationship Between Event Organizers and the Public Health Departments as New Players has not yet Settled Properly

As outlined above, the approval of events is regularly a responsibility of the municipal public order office. It is carrying out this task since ages in which it could accumulate expertise, especially in the assessment of “classic” safety and security risks and their technical, organizational or personal countermeasures. When expertise is missing, the public order office may involve specialized local agencies (see Fig. 2) for their assessment or opinion of a special issue (e.g. fire protection, public health, etc.). As infection prevention was hardly an issue in the past, public health authorities played only a minor role in the assessment process. Now that they entered the event stage as a key player with formal legitimacy, they face problems of acceptance as event organizers question their expertise and experience regarding the event sector:

“Our responsible approval authority is the public order office in conjunction with, or I’ll rather say, with the professional advice of the public health office. The people with whom we now discuss and talk day in and day out had no idea at all about major events. […] So of course, there are people at the public order office who have to deal with large events when they take place in public space. However, to consider an event under this aspect, the infection protection for event visitors, was actually technically not possible for these people at all. They first had to get to grips with it very deeply. It was a very big task for us to create a relationship of trust and to simply say: ‘Okay, we can agree to certain things and other things that might sound totally logical to someone from a health department are simply totally unrealistic.’" (Organizer 3, item 16)

The organizers therefore claim a certain power of definition vis-à-vis the public health authorities with regard to what is feasible and sensible in the context of large events. In this way, they make margins of manoeuvre possible for themselves, insofar as specifications appear to be too strict or unusual to them.

Different perceptions of the role of approval authorities in the re-opening process also shine through in this statement of a public order office director:

“I see the biggest challenges in the hygiene concepts that have to be submitted. They have to be countersigned by the health authorities. At least the [CITY] health authority is relatively restrictive in its approach. That is where I see the big problems. […] I really think our health department will be the biggest hurdle.” (Approval authority 1, item 11)

Since major events started to re-open in Germany, it has become customary that approval authorities demand so-called “hygiene concepts” from event organizers which are forwarded to the health authority for checking. No other actor feels responsible or knowledgeable enough to co-evaluate hygiene concepts (Approval authority 1, item 17; AOS 1, item 23; AOS 2 and 3, item 38). However, when it comes to assessing practical implementation, the health department drops out: The formal increase in importance and tasks has not been accompanied by a similar growth in work force. In our observations, we registered the attendance of several key stakeholders before and during an event (in preparatory meetings and inspections), while the health department did not show up (sports event 1, music festival 1, music festival 2). Event organizers know this professional weakness and therefore create sophisticated hygiene concepts as *facades* *of* *legitimacy*.

In summary, health departments are strongly oriented towards political guidelines, perceived by others as lacking a real feeling for and understanding of the “event” setting in general and its structures and processes. In order to be on the legally safe side, they tend to make maximum demands in terms of safety instead of selecting what makes sense. Due to the powerful gatekeeper function of health authorities in the approval process, their requirements and expectations must be met in the concept (on paper), even though they are not realistic or feasible. The adherence to self-imposed standards in these hygiene concepts declines over time if a lack of administrative supervision by health departments is felt by organizers during event conduction. Overfulfilment on paper serves as a strategy by organizers in order to please authorities during the event approval process. In this respect, legitimacy facades are already built up in the hygiene concepts, since organizers may be aware that the health offices will not check what is actually implemented at the event due to their capacity problems. The danger of organizational legitimacy facades grows (see above).

### Delegation of Pressure

Our research took place at the local level, which is where COVID-19-related health safety measures are implemented in accordance with rules made elsewhere (mainly federal state level). It is on the local level where stakeholders get together, negotiate, and finally decide on the approval of an event.

The local government and in particular its specialized authorities are in a position in which they have to safeguard the compliance to COVID-19-related rules and regulations that have been imposed by higher levels. This lets the executive branch—administration as well as public safety agencies—find themselves in a sandwich position between the rule of law and an implicit political will. Specialized authorities involved in the approval process report their notion of political pressure to re-enable the conduction of major events whenever possible. In the perspective of an interviewed public safety officer, local politics support a swift re-opening of major events in order to foster social cohesion and to get good social processes going again:

“If major events shall take place, politicians will always want to support them. Because that helps to get good social processes and social interaction going again, which is also very important for cohesion.” (AOS 1, item 19)

“It’s a political question: A fundamental decision about whether you want a new start and under which conditions can actually only be made politically. In the end, this decision is the basis for any action by the approving authorities. Therefore, a [municipal] health authority will not approve or prohibit an event despite these regulations at the political level, but will be guided by them.” (AOS 2 and 3, item 35)

Approval authorities as well as AOS also describe the momentum behind the political will as ‘impulses’ that can hardly be resisted, or even as “insane pressure”. This results in the impression that it is not the technical approval scheme alone that drives the approval process:

“I would never claim that the approval authorities at the municipal level are completely free in their decisions. Of course, there are political impulses. And there has to be a lot in the balance for them to go against these political impulses.” (AOS 1, item 25)

“I can see that many events are also politically desired, of course. And at the moment there is simply an insane pressure behind it that a return to the normalization of events is actually desired.” (Health authority 1, item 5)

There seems a certain domino effect starting with politics—approval authorities follow the political will, and public safety agencies such as police and firefighters follow the approval agencies:

“They [the approving authority] set the standard, so to speak. If they indicate – and I will put it quite casually—‘we don’t take it very seriously, we don’t want to bully the citizen too much, infection protection takes a back seat’, then we also agree with this vote, because for us this is of course the specialist authority that we have to align ourselves with. Anything else would in any case lead to us causing more problems for society, I believe, and would exceed our resources. We are not equipped to take over the entire regulatory area.” (AOS 1, item 11)

At this level, the strong legal orientation along legal requirements and a certain political bondage are evident on the part of the approval authorities. This gives the impression that some approval authorities may want to act in a legally secure and politically compliant manner vis-à-vis the event organizers. Such authorities seem to be less concerned with the acceptability, meaningfulness and feasibility of measures. In addition to a particularly extensive conceptualization, organizers must also deal with irregular changes in a dynamic pandemic situation. However, as illustrated in the section below, available adjusting screws for orientation also remain unused.

## Outlook/Discussion

With the findings presented in this article, we aimed to provide a case study of how the changing COVID-19 enviorment influences interorganizational relationships, mutual expectations, and organizational strategies in the crisis from the perspective of event organizers.

*Event* *organizers* are required to follow various logics in order to get their events legitimated and to be allowed to hold them. In order to meet expectations of the approval authority, respectively, the health authority, they aim to conceptualize and implement all required measures (security and hygiene), even if they know that these are neither implementable nor comprehensible. They must coordinate with approval agencies and explain deviations from maximum requirements and/or using the remaining margins of manoeuvre. Sometimes this creates facades of legitimacy, since many things are listed or represented in event structures, but are not necessarily translated into actual actions. In coordination with public safety and security organizations, event organizers need to check unwanted interactions between “new” hygiene measures and “classic” safety measures (e.g. face coverings were regularly banned in football stadiums for CCTV surveillance of problematic individuals which was overthrown by the requirement to wear a mask). The organizer has to respond to the visitors (audience wants certain measures: “security theatre”), as s/he wants them to come to the events; considerations of economic efficiency: measures must not be too expensive (weighing safety or hygiene, if necessary). The whole approval process has become very demanding, while at the same time, event conduction and customer behaviour are uncertain. Small and low/non-profit event organizers may avoid the effort and refuse from planning at all. The event sector concentrates on the bigger players.

But also the roles of approval authorities and authorities and organizations with safety and security tasks changed. *Approval* *authorities* strive for legal protection and legitimacy. They cannot decide or approve beyond the period of validity of the rules in force at the time. This temporal logic of the authorities clashes with the need for long-term planning of major events. *Authorities* *and* *organizations* *with* *safety* *and* *security* tasks also strive for legal protection and legitimacy (see above). However, their focus is on safety, not at all on hygiene. However, they may experience a higher workload nowadays due to facilitating and implementing hygiene measures and hygiene communication for event-goers.

In order to complement the perspectives of the event organizers with the event-goers’ attitude towards hygiene compliance at large events, we are currently reflecting on results of a representative German-wide survey on audience perspectives as well as our event observations to assess which measures are acceptable and enforceable. Eventually, a planning and decision support tool for both event organizers and approval authorities will be created in order to scientifically support both sides in the event approval process under pandemic circumstances and enhance planning reliability.

In summer 2022, when this article is finalized and revised, COVID-19 is mainly unregulated in the event sector, as restrictions have largely been lifted in Germany. Of all the lessons learned under pain, only a few are still put into practice by responsibly acting event organizers who do not like to see their staff, contractors and customers at risk. However, this unfortunately fits into the larger picture of the event sector, which has repeatedly and for various reasons shown itself to be incapable of collective self-regulation. It can therefore be assumed that this group of actors, without any significant initiative of its own, must comply to potentially recurring external demands in order to gain legitimacy and be allowed to organize events.

## Footnotes

1 Interview quotations are referenced in brackets: “Stakeholder group” followed by an ordinal number and the number of the respective paragraph in the interview transcript.
